# Geographical Position of Baltimore

**Published:** 1880-08

**Authors:** 


Geographical Position of Baltimore.— When we contemplate the present situation of affairs, and cast our thoughts but a brief space into the future of our city, with its now almost limitless resources for greatness and grandeur in nearly every direction in which the cultivated human intellect can reasonably hope for the consummation of earthly happiness and enjoyment, we feel indeed, "our lines have been cast in pleasant places." Standing as Baltimore does, almost upon the famous Mason & Dixon's line, she occupies neutral ground, so to speak, between the two great geographical and social, if not political divisions of North and South; the extended domain of which embraces thousands of miles of territory on the Atlantic ocean and gulf of Mexico, with every desirable variety of climate to be found within twenty degrees of north latitude. At the head of the Chesapeake bay, with railways stretching their iron arms in every direction, and sending great arteries of commerce to the vast and prolific west, and receiving its bounteous products in return. Within less than an hour's ride from the capitol of the Republic, and already possessed of educational, scientific, charitable and other institutions, the most of which any city or community need not be ashamed, but indeed be proud. Notably among these being the Johns Hopkin's University, the University of Maryland, and Baltimore City College. The literary and scientific departments of the former already operating well, and should the medical department be wisely organized, with the unequalled hospital advantages and facilities at its command, it *need not* have a peer in the world? The University of Maryland has a very successful law school, and should the medical school be remodeled, as contemplated, with its present material, there is no good reason why it should not be classed among the best of such institutions in the country. Time and space would fail us to speak of the Peabody Institute, the Maryland Institute, the Hopkin's and Mercantile Libraries, etc., hence we can only say in conclusion that the foregoing, and those already recorded in these pages, together with the *vim* and *energy* that can and will continue to be brought to bear, will all be utilized in rendering the *Independent Practitioner* acceptable to every section of our country. In its conduct and management it will know no north, no south, no east, no west; but be the exponent its geographical position requires, of legitimate medicine throughout the vast continent on which we live.

ERRATA.—Several errors occurred in the last form printed for the July issue of this Journal, owing to our inability to revise the proof before it was compelled to go to press; fortunately they are quite slight, and the reader will readily correct them with the eye as he passes over them. While typographical errors are almost inseparable from a serial of this character, it is our intention to render this as free from such blemishes as possible, and therefore trust our readers will pardon the short comings in our last.

Our Dental Patrons will please excuse any want of miscellany in their department of the Journal, as our junior, who has exclusive control of it, is absent from the city for recuperation and relaxation from business for a little while.

